# The Harvard Beat Assessment Test (H-BAT): a battery for assessing beat perception and production and their dissociation

**DOI:** 10.3389/fnhum.2013.00771

**Published:** 2013-11-26

**Authors:** Shinya Fujii, Gottfried Schlaug

**Affiliations:** Department of Neurology, Beth Israel Deaconess Medical Center and Harvard Medical SchoolBoston, MA, USA

**Keywords:** rhythm, beat, meter, synchronization, beat-deafness, battery, dissociation

## Abstract

Humans have the abilities to perceive, produce, and synchronize with a musical beat, yet there are widespread individual differences. To investigate these abilities and to determine if a dissociation between beat perception and production exists, we developed the Harvard Beat Assessment Test (H-BAT), a new battery that assesses beat perception and production abilities. H-BAT consists of four subtests: (1) music tapping test (MTT), (2) beat saliency test (BST), (3) beat interval test (BIT), and (4) beat finding and interval test (BFIT). MTT measures the degree of tapping synchronization with the beat of music, whereas BST, BIT, and BFIT measure perception and production thresholds via psychophysical adaptive stair-case methods. We administered the H-BAT on thirty individuals and investigated the performance distribution across these individuals in each subtest. There was a wide distribution in individual abilities to tap in synchrony with the beat of music during the MTT. The degree of synchronization consistency was negatively correlated with thresholds in the BST, BIT, and BFIT: a lower degree of synchronization was associated with higher perception and production thresholds. H-BAT can be a useful tool in determining an individual's ability to perceive and produce a beat within a single session.

## Introduction

One of the definitions of *rhythm* is the pattern of time intervals in a stimulus sequence, and *beat/pulse* refers to a series of regularly recurring psychological events that arise in response to the musical rhythm (Cooper and Meyer, 1960; Large, 2008; Grahn, 2012). *Meter* refers to the temporal organization of beats, in which some beats are perceived as more salient than others (Cooper and Meyer, 1960; Lerdahl and Jackendoff, 1983; Large, 2008; Grahn, 2012). For example, duple meter refers to a pattern of alternating strong (S) and weak (w) beats (SwSwSw…), whereas a triple meter refers to a pattern of a strong beat followed by two weak beats (SwwSwwSww…) (Ellis and Jones, 2009). The beat acts as a catalyst in stimulating spontaneous timely movements such as tapping our feet or nodding our heads (Chen et al., 2006). The ability to perceive, produce, and synchronize with the beat is thought to be widespread across individuals (Grahn and Shuit, 2012). Anecdotally, however, while some individuals are indeed very good at tapping, clapping, or dancing with a beat, others appear to have “no sense of rhythm” and cannot detect or synchronize with the beat of a musical rhythm and might be referred to as “beat-deaf.” In this paper, we describe a test that might reveal such individual differences in beat-processing ability using a series of subtests assessing beat perception and production.

Recently, Phillips-Silver et al. (2011) reported a case of beat-deafness. They defined the individual as beat-deaf by showing that his perceptual discrimination of meter (duple or triple) in piano patterns was poor compared with a normal population as well as showing that his bouncing and tapping movements were not phase-locked with a musical beat (Phillips-Silver et al., 2011). Interestingly, the beat-deaf individual had an impairment only in beat processing without any impairment of pitch processing (Phillips-Silver et al., 2011). This indicates that beat-deafness could present a new form of a congenital or acquired disorder related to time and not to pitch. Previous neuroimaging studies have shown that the pitch-processing disorder in tone-deafness (or congenital amusia) is related to abnormalities in temporal and frontal brain regions as well as the connections between these regions (Hyde et al., 2007; Mandell et al., 2007; Loui et al., 2009; Hyde et al., 2011). The neural underpinnings of beat processing, on the other hand, are still relatively unexplored.

In studying sensorimotor synchronization, two distinct internal processes have been assumed to account for the observed behaviors (see Repp, 2005, for a review). For example, in a classic model by Wing and Kristofferson (1973a,b), the time variability of rhythmic tapping is assumed to consist of “clock” and “motor” variances. The former is a variance included in the central or internal-timekeeper process issuing tap commands, and the latter is a variance included in the peripheral process executing the command into action (Wing and Kristofferson, 1973a,b). A linear error-correction model proposed by Mates (1994) also assumes two processes to explain sensorimotor synchronization. One is called “period correction” which modifies the period of an internal timekeeper, and the other is called “phase correction” which is considered to be a local adjustment to the interval generated by the timekeeper while it leaves the period of the timekeeper unaffected (Mates, 1994). Repp and colleagues have tested these assumptions experimentally and have shown that the period correction requires conscious awareness and attention while the phase correction is largely unconscious and automatic (e.g., Repp, 2001; Repp and Keller, 2004; Repp, 2005).

Tone-deafness studies have also assumed two distinct internal processes during pitch processing (Loui et al., 2008, 2009). For example, the individuals, who cannot consciously perceive pitch directions, can reproduce pitch intervals in correct directions, showing that there is a dissociation between pitch perception and production (Loui et al., 2008). Interestingly, Loui et al. (2009) found that the volume of the superior arcuate fasciculus (AF), a fiber tract connecting temporal and frontal brain regions, was a significant predictor of conscious pitch discrimination ability, whereas the inferior AF volume predicted the degree of perception-production mismatch. These findings were thought to support an auditory “dual-stream” hypothesis (Hickok and Poeppel, 2004; Griffiths, 2008), which assumes that auditory information is processed in two distinct channels: (1) a ventral stream that is concerned with conscious perception and (2) a dorsal stream that enables the connection with the motor system for automatic motor production (action) in response to auditory stimuli.

Considering these dual-process models, an interesting question in the study of beat perception and production is whether a dissociation exists between them. In a case study of beat-deafness by Phillips-Silver et al. (2011), a clear dissociation between beat perception and production could not be found. The beat-deaf individual failed to move the body in synchrony with the musical beat and could not discriminate meter (duple/triple) perceptually (Phillips-Silver et al., 2011). However, one would still expect to find individuals who have normal beat perception but cannot produce a beat, or vice versa. Sowinski and Dalla Bella (2013) recently reported the presence of two individuals who showed poor synchronization to music without any impairment of rhythm perception (i.e., they were able to discriminate differences in time-interval patterns, see S1 and S5 in their paper). Although it has not been reported so far, one might also expect to find a reverse dissociation: poor rhythm perception without any impairment of production. If these cases exist, another interesting question would be what kind of neural mechanisms would underlie these dissociations. However, the study of beat perception and production and that of its disorder, beat-deafness, is still in its infancy and more research is needed to address these questions. To advance the study of beat perception and production and to examine the psychophysical underpinnings of beat-deafness, it is important to develop a battery, which (1) can be performed within a reasonable period of time sampling an underlying population and (2) is able to identify dissociations between beat (rhythm) perception and production.

The Montreal Battery for the Evaluation of Amusia (MBEA) (Peretz et al., 2003) is currently a widely used battery of tests to screen perceptual problems in pitch, rhythm, and meter. The MBEA consists of six subtests (referred to as contour, interval, scale, rhythm, meter, and memory tests) and takes about 1.5 h of testing. In the rhythm subtest in the MBEA, participants hear two short sequences of piano sounds and are asked to discriminate whether the tone-interval patterns are the same or different. In the meter subtest, they hear one sequence of piano sounds and are asked to discriminate whether the underlying meter is duple (march) or triple (waltz) (Peretz et al., 2003). These rhythm and meter subtests have been used in recent studies of beat-deafness to detect perceptual deficits in rhythm and meter processing (Phillips-Silver et al., 2011; Sowinski and Dalla Bella, 2013). However, the pitch-changing piano patterns that are used for the stimuli in the rhythm and meter subtests also tap into pitch-processing abilities (Foxton et al., 2006). Furthermore, a functional Magnetic Resonance Imaging (MRI) study showed that sensorimotor mapping networks were sensitive to both pitch and temporal structure in music (Brown et al., 2013), suggesting that pitch and rhythm processing could interact with each other at neural and behavioral levels. The use of monotonic or woodblock sounds can avoid such confounds. Actually, those types of stimuli were used in the Macquarie monotonic rhythm test (Thompson, 2007), musical ear test (Wallentin et al., 2010), and profile of music perception skills (Law and Zentner, 2012). However, these tests only assess perceptual but not production abilities.

The beat alignment test (BAT) was proposed to assess both perception and production (synchronization) abilities in beat processing (Iversen and Patel, 2008). The BAT requires the participants to judge whether or not beeps superimposed on musical excerpts were on the beat in the perception task, and requires participants to tap in synchrony with the beat of music in the production task. The BAT uses exactly the same set of musical excerpts in both perception and production tasks. This is a good approach when one is interested in investigating two internal processes (i.e., perception and production) using the same external auditory stimuli. The use of musical excerpts is also reasonable for simulating the musical activity of daily living, but it also leads to a confound: The perception of musical excerpts does not only include beat perception but also pitch, melody, harmony, and timbre perception as well touches on memory and emotional processes (Tramo, 2001; Koelsch, 2012). These factors may interact with each other (e.g., Foxton et al., 2006; Brown et al., 2013) and affect beat perception and production in different ways. An alternative approach is to use psychophysics with controlled auditory stimuli. This approach has been used successfully in establishing neural correlates of observed auditory behaviors (e.g., Loui et al., 2009; Grube et al., 2010; Mathys et al., 2010; Teki et al., 2011b).

A hybrid approach is to use both musical excerpts and controlled stimuli to test the beat-processing ability. The Battery for the Assessment of Auditory Sensorimotor Timing Abilities (BAASTA) (Farrugia et al., 2012) applied this approach and integrated many tasks from previous studies: duration-discrimination task (Buhusi and Meck, 2005), anisochrony-detection task with tones and musical sequences (Hyde and Peretz, 2004), the BAT (Iversen and Patel, 2008), unpaced-tapping task (Drake et al., 2000), paced-tapping task with metronome and musical stimuli (Repp, 2005), synchronization-continuation tapping task (Wing and Kristofferson, 1973a,b), and an adaptive-tapping task (Repp and Keller, 2004; Schwartze et al., 2011). The BAASTA showed that the patients with Parkinson's disease failed to tap with musical excerpts but also could not adapt to a temporal change in the metronome. On the other hand, the patients were not impaired in the perceptual tasks to discriminate duration and anisochrony of tone sequence (Farrugia et al., 2012). The authors suggested that the absence of deficits in perceptual tasks could be due to an effect of dopaminergic replacement therapy. Although the use of musical excerpts and controlled tone stimuli has advantages in determining the normal and impaired time-processing abilities, a major issue of the BAASTA is that the test duration is very long (2.5–3 h in total) and that BAASTA does not use the same set of auditory stimuli for perception and production tasks (e.g., discrimination thresholds were measured in the perception tasks while tapping accuracy and variability were measured in the production tasks). To assess the dissociation more directly, it might be better if one could assess both perception and production thresholds from the same set of auditory stimuli using a psychophysical technique as was done in previous studies of tone-deafness (Loui et al., 2008, 2009).

Building upon these prior approaches to test beat perception and production, we developed the Harvard Beat Assessment Test (H-BAT), a battery of tests to assess beat perception and production abilities. We will show that the H-BAT can be performed within a reasonable period of time. The H-BAT applies the hybrid approach using both musical excerpts and psychophysically-manipulated woodblock stimuli to test beat-processing abilities of individual participants. It measures both perception and production thresholds from the same set of auditory stimuli via psychophysical adaptive stair-case methods. Our first goal with the H-BAT was to establish objective measures and cut-off scores in a sample of the local population with the ultimate goal to have criteria to identify individuals who are performing below the cut-off scores and could be identified as beat-deaf. The second goal was to investigate the distribution of perception and production thresholds to explore the possibility of a dissociation between the two thresholds.

## Methods

### Participants

We performed the H-BAT on thirty healthy participants (15 males and 15 females) who had no history of neurological or psychiatric disorders. The mean age was 27.2 ± 7.2 years (range: 21–58). We evaluated the handedness by the Edinburgh Handedness Inventory (Oldfield, 1971). The mean laterality quotient was 76.7 ± 36.4 (ranged from −40 to +100, two of them were left-handers). The participants had a range of musical experience: some had no experience of practicing musical instruments whereas some practiced or had been practiced playing keyboard, string, wind, and/or percussion instruments. The mean duration of musical training was 11.8 ± 8.3 years (range: 0–31). The mean (±*SD*) accumulated hours of training estimated from a musical-background questionnaire was 9607 ± 9375 (range: 0–33945 h). This study was approved by the Institutional Review Board of Beth Israel Deaconess Medical Center.

### Test design

**Figure 1:**
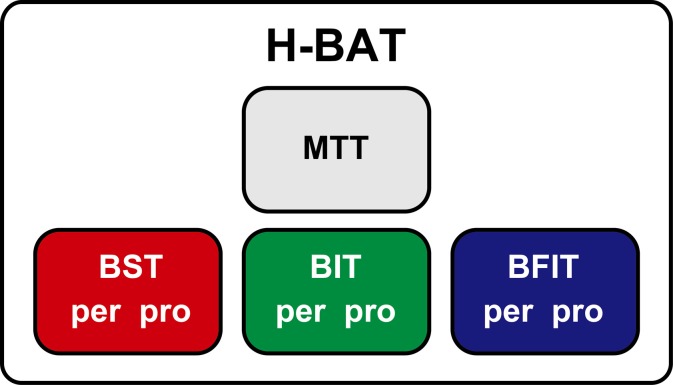
**A schematics of the Harvard Beat Assessment Test (H-BAT)**. H-BAT consists of four subtests; music tapping test (MTT), beat saliency test (BST), beat interval test (BIT), and beat finding and interval test (BFIT). The BST, BIT, and BFIT have a perception (per) and production (pro) part each.

The H-BAT consisted of four subtests (Figure 1); music tapping test (MTT), beat saliency test (BST), beat interval test (BIT), and beat finding and interval test (BFIT). The MTT measures the individual ability to synchronize the tapping movement with the musical beat using musical excerpts. The BST, BIT, and BFIT consist of two parts measuring both perception and production thresholds using psychophysically-manipulated woodblock stimuli (Figure 1).

### Apparatus

**Figure 2:**
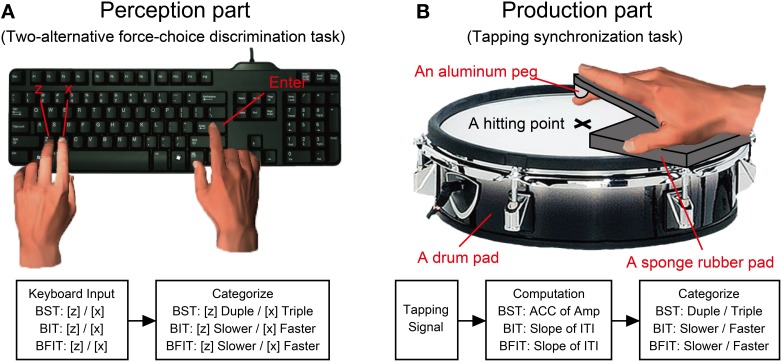
**A schematics of experimental apparatus and procedure. (A)** Participants responded using a keyboard in the perception parts of Beat Saliency Test (BST), Beat Interval Test (BIT), and Beat Finding and Interval Test (BFIT). **(B)** In the production parts, participants tapped on a drum pad using their index finger. We computed auto-correlation coefficients (ACC) of tapping amplitudes and slopes of the regression lines in the inter-tap intervals (ITIs). The behavioral responses were categorized based on the computation from the tapping signal.

The H-BAT was implemented on a laptop (Windows 7, 64 bit version, CPU@2.40GHz, 8.00GB RAM, HP) using Matlab 7.5.0 software (Mathworks) with the data-acquisition toolbox. We used an USB external keyboard (DELL) and an electric drum pad (10 inches in diameter, PD-105, ROLAND) to record the participant's response (Figure 2). A black cushioning pad, which was made from ethylene-propylene rubber (10 mm in thickness, WAKI, Japan), was placed on the drumhead to rest the hand palm comfortably on the drum pad (see Figure 2B). A previous study showed that hitting the tapping surface with a hard material (e.g., a drumstick) was better to get sharp signals compared to hitting with a soft finger's pad (Fujii and Oda, 2009). To make the tap signal as clear as possible when hitting the drum pad, we attached another cushioning pad which tip was made of an aluminum peg (BE-385, NIKAYA, Japan) along with the participant's index finger (see Figure 2B). The signal from the drum pad was sent to MIC/INST input of an audio/MIDI interface (US-600, TASCAM). The signal of the auditory stimulus was split into two signals: one was provided to the participants via headphones binaurally (Quiet Comfort 15, Acoustic noise cancelling headphones, BOSS), and another was sent to MIC/INST input of the audio/MIDI interface. The noise cancelling headphones were used to reduce auditory feedback from the self-generated sound of the finger taps. The headphones actively cancelled the sound of the participant's finger taps in addition to delivering the stimuli. The auditory stimuli were played at the sampling frequency of 48000 Hz. The signals of the drum pad and auditory stimulus were synchronized and recorded with a sampling frequency of 8000 Hz.

### Subtests

#### Hearing test

To control the amplitude levels of the auditory stimuli across the participants, a short hearing test was performed prior to the H-BAT. We played pure tones with three frequencies (1000, 2000, and 4000 Hz). The participants responded whether or not they heard the tone by pressing either the *Z* (yes) or *X* (no) buttons, and had to press the *Enter* after each of these key-presses. The test started with the tones whose sound pressure level was 42 decibels (dB) (as measured by a sound pressure meter, RADIOSHACK). Every time when the participants responded yes/no, the peak amplitude of tone was changed to be 5 dB smaller/larger. Every time the answer changed from “yes to no” or “no to yes,” the amplitude at which this occurred was recorded as a turnaround point. The test was completed once a participant had gone through five turnaround points for each frequency. We discarded the first turnaround point to avoid a warm-up effect for each frequency. We then calculated the average across the turnaround points and defined it as the participant's hearing threshold in this study. The order of the three frequencies was randomized across the participants.

#### Music tapping test (MTT)

Previous studies reported that the experimental paradigm investigating movement synchronization with a musical beat was appropriate to assess possible beat-deafness in individuals (Phillips-Silver et al., 2011; Sowinski and Dalla Bella, 2013). Thus, we considered that the degree of synchronization with the beat in music could be a direct measure for determining deafness to a particular beat in certain individuals. We designed the MTT based on this idea.

**Figure 3:**
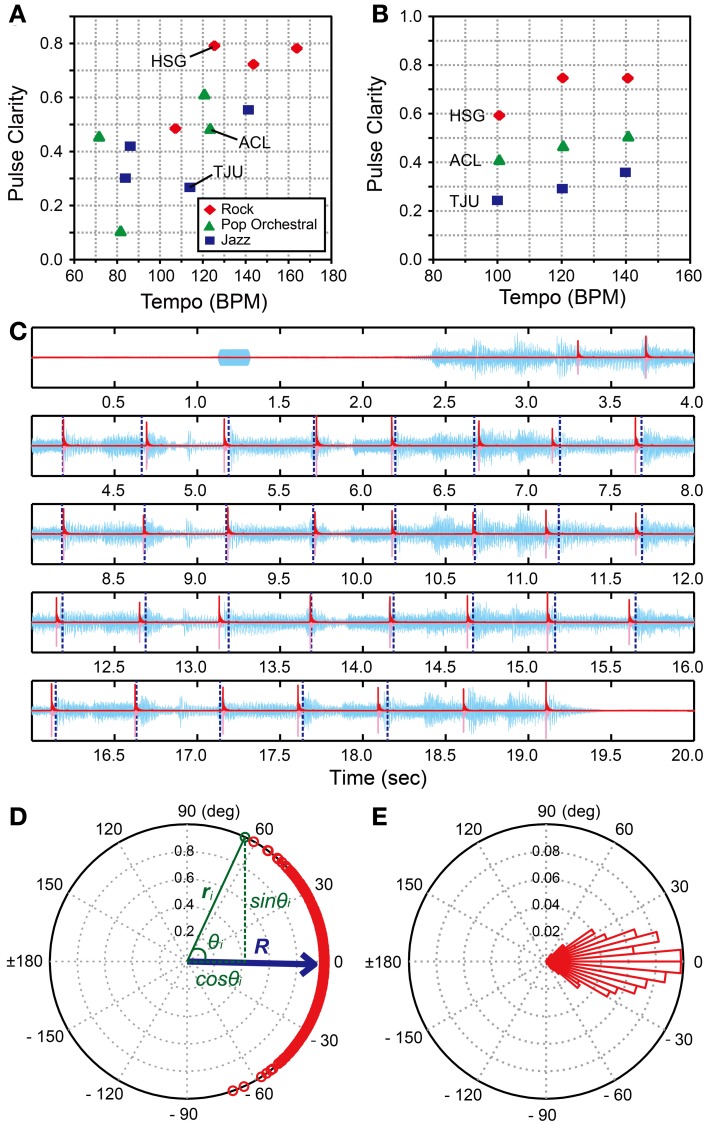
**Music tapping test (MTT). (A)** BPM—pulse clarity plots of 12 musical excerpts used in the beat alignment test (BAT) (Iversen and Patel, 2008). We selected 3 excerpts (i.e., HSG, Hurts So Good by J. Mellencamp; TJU, Tuxedo Junction by Glenn Miller; and ACL, A Chorus Line by Boston Pops) for the Harvard Beat Assessment Test (H-BAT). **(B)** BPM—pulse clarity plots of 9 musical excerpts used in the H-BAT. **(C)** An example of recorded tapping signal (pink), its envelope (red), and audio wave during playing HSG (light blue). Vertical blue dashed lines represent the quarter-note beat timings (Iversen and Patel, 2008). **(D)** A plot of relative phases between tap onsets and beat timings (*n* = 486) on a unit sphere across 18 trials (9 stimuli × 2 trials) in a participant (red). The resultant vector (*R*) is shown as blue arrow. **(E)** A histogram of the relative phases. Each bin has a range of 5°.

We used the 3 musical excerpts from the BAT (version 2, Iversen and Patel, 2008, the materials were downloaded from: http://www.nsi.edu/~iversen/); *Hurts So Good* (*HSG*) by *J. Mellencamp* (rock style, duration = 14 s), *Tuxedo Junction* (*TJU*) by *Glenn Miller* (jazz style, duration = 16 s), and *A Chorus Line* (*ACL*) by *Boston Pops* (pop-orchestral style, duration = 14 s). These excerpts were selected based on the following steps. First, we calculated the pulse clarity measures of all the 12 musical excerpts in the BAT (Iversen and Patel, 2008) using the “mirpulseclarity” functions in the MIR toolbox in Matlab (Lartillot and Toiviainen, 2007; Lartillot et al., 2008b). Note that the pulse clarity measure is considered to be correlated with the listeners' subjective ratings of the pulse clarity in a given musical excerpt (Lartillot et al., 2008a). That is, the lower the measure, the more ambiguous the pulse becomes. It is calculated from autocorrelation function of the envelope of audio waveform (Lartillot et al., 2008a). We also calculated the beats per min (BPMs) of the 12 excerpts using the beat-timing data reported in the BAT (Iversen and Patel, 2008). Second, from the BPM—pulse clarity plot of the 12 excerpts (Figure 3A), we selected 3 excerpts (HSG, TJC, and ACL) from the rock, jazz, and pop-orchestral music styles with pulse-clarity measures that were around 120 BPM. Next, we used Audacity (version 2.0.2) and changed the tempi of HSG, TJC, and ACL to be 100, 120, and 140 BPMs (i.e., slow, medium, and fast tempi) by using the “Change Tempo” function which changes tempo (speed) of the audio without changing pitch. That is, we created 9 musical stimuli in total; 3 BPMs × 3 styles of music (Figure 3B and Supplementary Material). Thus, the stimuli in H-BAT were distributed in a controlled way in terms of the BPM—pulse clarity plot. The peak amplitudes of the musical excerpts were normalized to be 25 dB larger than the participant's hearing thresholds. A 1000-Hz pure tone was added 1-s before the musical excerpt in order to signal the beginning of the trial (Figure 3C). The pure tone had 200-ms duration with a 2-ms rise-fall time. The peak amplitude of the pure tone was 20 dB larger than the hearing threshold.

The task was to tap the quarter-note beat underlying the musical excerpts (see Figure 3C). The experimenter demonstrated how to tap the quarter-note beats and played HSG, ACL, and TJU at their original tempo (125.4, 123.5, and 114.0 BPMs, respectively) as practice trials. If the participants tapped not the quarter-note beats but the half-, eighth-, or syncopated-fourth-note beats, they practiced the excerpt again until they understood how to tap the non-syncopated quarter-note beats. The participants were also asked to close their eyes during the data recording to prevent visual feedback. The 9 stimuli were repeated twice for each participant; a measurement of the task consisted of 18 trials in total. The order of the stimuli was randomized across the participants.

We calculated an envelope of the tapping signal (red lines in Figure 3C) by using the Hilbert transform in order to detect the tap onset (see, Fujii et al., 2011). We defined the onset as the time at which the amplitude exceeded 10% of the maximum amplitude of the each tap. We used the data of quarter-note beat timings from the BAT (version 2, Iversen and Patel, 2008) (see, vertical dashed lines in Figure 3C). To quantify the degree of tapping synchronization with the beat timings, we calculated the relative phase between the tap onset and the beat timing (see also, Fujii et al., 2010). The value of relative phase is equal to 0° when the participant's tap was perfectly synchronized with the beat timing and ±180° when the tap time was located perfectly in a middle between the beat timings. The value is negative when the tap precedes the beat timing and positive otherwise. We pooled the relative-phase data across the 18 trials (486 beats in total). A typical example of the relative-phase distribution is shown in Figures 3D,E. Note that the relative phase (θ) is expressed in degrees.

**Figure 4:**
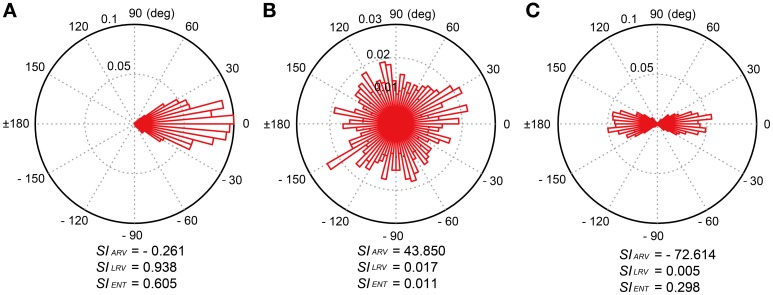
**Examples of relative-phase distribution and calculated synchronization indices (*SI*s)**. There were three *SIs*; (1) angle of resultant vector (*SI*_ARV_), (2) length of resultant vector (*SI*_LRV_), and (3) entropy of relative phase distribution (*SI*_ENT_). **(A)** Uni-modal relative-phase distribution around 0 degree. **(B)** Random distribution across all the degrees. **(C)** Bi-modal distribution around 0 and 180 degrees. The *SI*_ENT_ is more sensitive to detect the difference between the random phase distribution and the mixtures of in-phase and anti-phase lockings compared to the *SI*_LRV_.

In order to quantify the properties of the relative-phase distribution in an individual, we introduced three *synchronization indices* (*SI*s); (1) angle of resultant vector (*SI*_ARV_), (2) length of resultant vector (*SI*_LRV_), and (3) entropy of relative-phase distribution (*SI*_ENT_). The *SI*_ARV_ is a measure of synchronization *accuracy* while the *SI*_LRV_ and *SI*_ENT_ are measures of synchronization *consistency*. To compute these indices, the data set of relative-phases (*n* = 486) was plotted on a unit sphere (red and green circles in Figure 3D). The x- and y-coordinates of a vector *r*_*i*_ correspond to cosine and sine of an *i*-th relative phase θ*_i_* (see green texts in Figure 3D). The resultant vector *R* was then calculated by

(1) $$R=\frac{1}{n}\sum_{i=1}^{n}r_{i}$$

where *n* = 486 in this study. The *SI*_ARV_ and *SI*_LRV_ in this paper correspond to the angle and length of the resultant vector *R*, respectively. The *SI*_ARV_ is negative when the taps tend to precede the beat timings and positive otherwise. The *SI*_LRV_ ranges from 0 to 1 and the value becomes higher when the participant taps consistently in a certain phase relative to the beat timings (see Figure 4A), the value becomes lower when the relative phases are distributed randomly (e.g., the participant randomly taps ignoring the beat timings, see Figure 4B). The *SI*_LRV_ was actually used in a previous study of beat-deafness (Sowinski and Dalla Bella, 2013), however, the measure becomes problematic when in-phase and anti-phase are mixed (Figure 4C). It is not sensitive to detect the difference between the random phase distribution and the mixtures of in-phase and anti-phase lockings (compare Figure 4B to 4C).

Thus, we introduced another measure of synchronization consistency using *Shannon entropy* (*SE*) (Shannon, 1948), which is defined as the average value of logarithms of the probability density function:

(2) $$SE=-\sum_{i=1}^{M}p(i)\ln p(i)$$

where *M* is the number of bins with non-zero probability and *p*(*i*) is the probability of the *i*-th bin. The bin ranged from −180° to +180° with a 5° increment step in this study (Figures 3E, 4). The *SI*_ENT_ in this paper was then calculated as

(3) $$SI_{\text{ENT}}=1-\frac{SE}{\ln N}$$

where *N* is the total number of bins (see Tass et al., 1998; Mase et al., 2005). The *SI*_ENT_ ranges from 0, when the spreading of relative phase is maximal (i.e., when all phases lie in different bins), to 1, when a *δ*-function like probability distribution is found (i.e., all phases lie in a single bin). That is, the larger the *SI*_ENT_, the stronger the phase of a participant's tap is locked to the beat timings. In contrast to the *SI*_LRV_, the *SI*_ENT_ is sensitive to detect the difference between the random phase distribution and the mixtures of in-phase and anti-phase lockings (see Figures 4B,C).

#### Beat saliency test (BST)

Phillips-Silver et al. (2011) showed that their beat-deaf participant failed to discriminate duple and triple meter, suggesting that the individual had difficulty in processing a hierarchical organization of alternating strong (S) and weak (W) beats (i.e., SwSwSw and SwwSwwSww). Chen et al. (2006) manipulated the hierarchical organization of beats via intensity accentuation and showed that the manipulation of beat saliency modulated tapping behavior as well as neural responses in the superior temporal gyrus (STG) and dorsal premotor cortex (dPMC): the more salient the accented beat became relative to the unaccented one, the more activation was found in auditory and dorsal premotor cortices. In addition, the functional connectivity between these two regions was modulated by the manipulation of beat saliency (Chen et al., 2006). These findings led us to consider that measuring the thresholds to perceive/produce beat saliency could be a useful measure to investigate the individual differences of beat processing. We designed the BST based on this idea.

**Figure 5:**
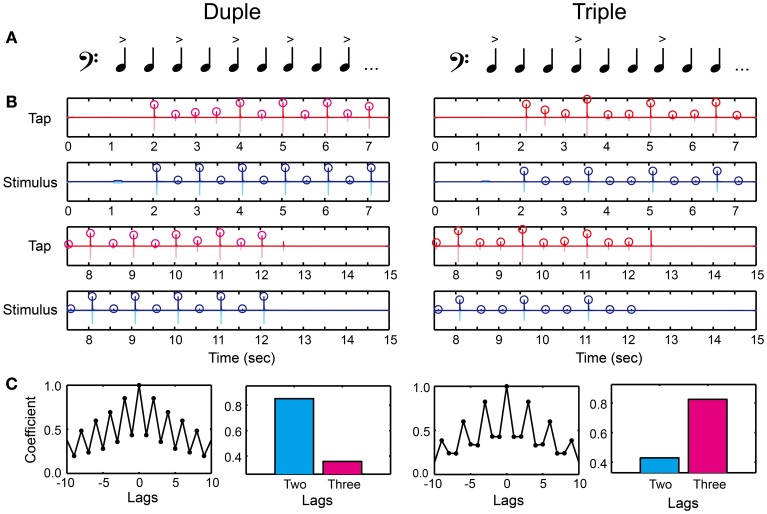
**Beat saliency test (BST). (A)** Duple or triple meter was created by making accents every two or three tones. **(B)** An example of recorded tap signal (pink), its envelope (red), recorded auditory stimulus (light blue), and its envelope (blue). Circles represent the detected peak amplitudes. **(C)** Auto-correlation coefficients calculated from the peak tap amplitudes. The participant's meter production was categorized as duple when the coefficient at two lag (cyan bar) was higher compared to that at three lag (magenta bar), whereas categorized as triple otherwise.

We created duple or triple meter by making intensity accents every two or three tones (Figure 5A and Supplementary Material). The tone sequence consisted of 1 pure tone and 21 woodblock tones (the audio waveform is shown as light blue in Figure 5B). We used the same woodblock tone used in previous studies (Chen et al., 2008a,b). The inter-stimulus interval (*ISI*) between the woodblock tones was 500 ms (i.e., 120 BPM) corresponding to the middle tempo of the musical excerpts in the MTT. We first calculated root mean square (RMS) of a woodblock-tone sequence without any accentuation (a flat sequence), which peak amplitude was 30 dB larger than the hearing threshold. The relative-intensity difference between the accented and unaccented tones was then modulated to create duple and triple meter, but the RMS intensity across the woodblock-tone sequence was kept the same as that in the flat sequence. A previous study showed that a 10-dB intensity difference between the accented and unaccented tones was enough to manipulate tapping behavior and brain responses in healthy normal participants (Chen et al., 2006). Thus, we started from 20-dB intensity difference between the accented and unaccented tones to make it clear enough. The relative-intensity difference was then manipulated according to the stair-case paradigm (see the section called “stair-case paradigm” below for the detail). In order to signal the beginning of the trial, the pure tone was played 1 s before the woodblock tones. The pure tone had 1000-Hz frequency and 200-ms duration with a 2-ms rise-fall time. The peak amplitude of pure tone was 20 dB larger than the hearing threshold, similar to what was done in the MTT.

There were two parts in the BST. The first was to discriminate the duple/triple meter perceptually by using a keyboard (Figure 2A). The second was to produce the meter by changing the tap amplitudes on the drum pad (Figure 2B). In the perception part, the participants placed the left middle finger on *Z* and the index finger on *X* buttons, and placed the right index finger on *Enter*. They had to decide which meter they were hearing by pressing either the *Z* (duple) or *X* (triple) buttons, and pressed the *Enter* after the decision. We asked them not to move any other parts of their body except for the finger movements to press the buttons (i.e., not bobbing the head or tapping the foot rhythmically) in order to measure the pure perception response. It is important to note that the perception part of the BST is different from simple volume detection task: the task cannot be performed only with the volume detection since the participant still have to categorize duple and triple by assuming the hierarchical organization of tones. For example, if the participant can detect the volume difference but cannot assume the hierarchical organization, the discrimination response will be random and thus the performance will be poor in the BST.

In the production part, we asked the participants to tap in synchrony with the woodblock tones using the index finger of the dominant hand on the drum pad (Figure 2B). We asked them to use metacarpal-joint movements. The task was to reproduce the duple or triple meter by changing their tap amplitudes. That is, the participants had to modulate the tap amplitudes according to the tone intensity. We detected the peak tap amplitudes during synchronizing with the woodblock-tone sequence (red circles in Figure 5B). We discarded the first six tap amplitudes to eliminate the start-up effect. We then calculated the auto-correlation function of the tap amplitudes (Figure 5C). A higher correlation-coefficient appeared at two (three) lags compared to at three (two) lags when the participants produced the duple (triple) meter. Thus, we categorized the participant's meter production based on the auto-correlation coefficients. It was categorized as duple when the coefficient at two lags was higher than that at three lags, while as triple otherwise (see bars in Figure 5C).

#### Beat interval test (BIT)

*Ritardando* (slowing down) and *accelerando* (speeding up) are basic notations indicating tempo change in music and are used to create or reduce tension, often when the end of a musical piece is approached. Anecdotally, performing gradual tempo changes accurately is quite demanding, particularly in ensemble playing. Shulze et al. (2005) studied synchronization of tapping with a metronome that smoothly changes tempo, from fast to slow (ritardando) or from slow to fast (accelerando), and showed considerable individual differences in the adaptation patterns. Madison (2004) also showed individual differences in the perceptual thresholds for detecting tempo change in a sequence which inter-onset intervals (IOIs) were linearly and continuously increased (ritardando) or decreased (accelerando). Moreover, the beat-deaf individual reported by Phillips-Silver et al. (2011) could not adapt to the gradual tempo change in music. These previous studies led us to consider that measuring the thresholds to perceive/produce a gradual tempo change could be a useful measure to investigate the individual differences of beat processing. The BIT was developed based on this idea.

**Figure 6:**
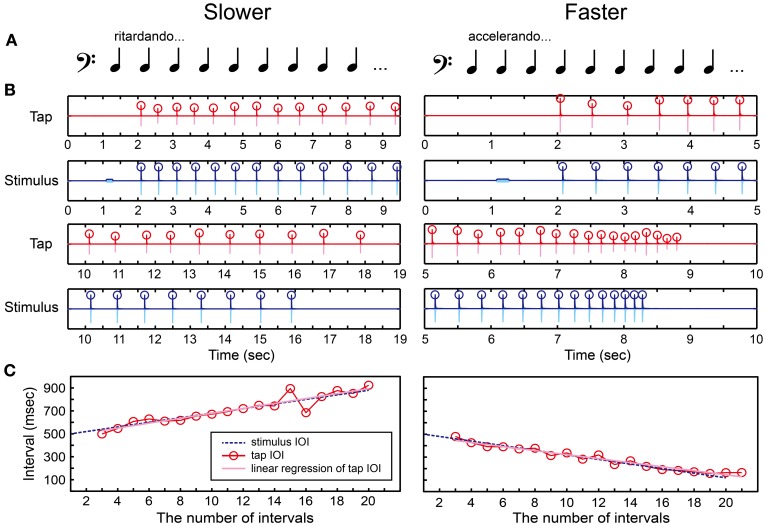
**Beat interval test (BIT). (A)** Two temporal-change patterns [slowing down (ritardando, slower) or speeding up (accelerando, faster)] were created by changing inter-stimulus interval (ISI) between woodblock tones. **(B)** An example of recorded tap signal (pink), its envelope (red), recorded auditory stimulus (light blue), and its envelope (blue). Circles represent the detected onsets. **(C)** The change of inter-onset interval (IOI) in the tap signal (red line and circles) and the auditory stimulus (blue dashed line). The slope of linear regression function of the tap IOI was calculated (pink line). The participant's beat-interval production was categorized as slowing down when the slope was positive whereas categorized as speeding up when negative.

We created a tone sequence consisting of 1 pure tone and 21 woodblock tones which tempo slowed down (*ritardando*) or sped up (*accelerando*) gradually (Figures 6A,B and Supplementary Material). The ISI between the woodblock tones was changed using the following equation:

(4) $$\{\begin{array}{c} ISI_{i+1}=ISI_{i}+d\;(\text{slower})\\ ISI_{i+1}=ISI_{i}-d\;(\text{faster}) \end{array}$$

where the first ISI equals to 500 (ms) and *d* is a constant. The tempo slows down if *d* is positive and speeds up otherwise. Madison (2004) showed that the perceptual threshold to detect continuous tempo change across 9 IOIs was around 10 ms. Thus, we started the parameter *d* from 20 ms to make it clear enough to notice the tempo change. The parameter *d* was then manipulated according to the stair-case paradigm (see the section called “stair-case paradigm” below for the detail). The pure tone was played 1 s before the woodblock tones, similar to what was done in the BST.

There were also two parts in the BIT. The first was to discriminate the temporal change perceptually responding with the keyboard (Figure 2A), and the second was to tap in synchrony with the tones and adapt to the temporal change by tapping on the drum pad (Figure 2B). In the perception part, the participants had to decide which temporal-change pattern they were hearing by pressing either the *Z* (slower) or *X* (faster) buttons, and pressed the *Enter* afterwards. Again, the participants were not allowed to move other parts of their body except for the fingers to press the buttons in order to measure the pure perception response.

In the production part, we asked the participants to tap in synchrony with the woodblock tones and to adjust their tapping tempo as precisely as possible according to the stimulus. We detected the tap onsets during synchronizing with the woodblock-tone sequence by using the same detection methods in the MTT (red circles in Figure 6B). We discarded the first-two and the last-two tap onsets to eliminate the start-up and end-up effects. We then calculated the slope of linear regression function of the IOIs (pink regression line in Figure 6C). A positive slope was observed in the tap-IOI time series when the participants produced the slowing-down beat whereas a negative slope was observed when speeding up. Thus, we categorized the participant's beat-interval production as slowing down when the slope was positive, while as speeding up otherwise.

#### Beat finding and interval test (BFIT)

Previous studies found that musicians are better at finding the beat or pulse in a pattern of time intervals compared to non-musicians (Chen et al., 2008b; Grahn and Rowe, 2009). It has been suggested that the superior ability of musicians to find the beat is associated with greater involvement in prefrontal cortex (Chen et al., 2008b) and increased connectivity between auditory and premotor cortices (Grahn and Rowe, 2009). These findings led us to consider that adding a beat-finding component into the BIT can be another important task to investigate the individual differences of beat processing. The BFIT was developed based on this idea.

**Figure 7:**
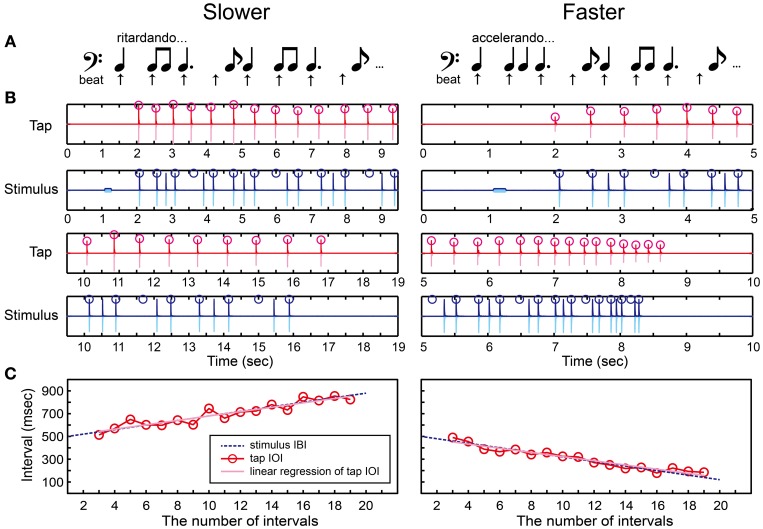
**Beat finding and interval test (BFIT). (A)** A rhythmic pattern was created by repeating a quarter note, two eighth notes, a dotted-quarter note, and an eighth note. The beat underlying the pattern (upper arrows) slows down (ritardando, slower) or speeds up (accelerando, faster). **(B)** An example of recorded tap signal (pink), its envelope (red), recorded auditory stimulus (light blue), and its envelope (blue). Red circles represent the detected tap onsets and blue ones represent the quarter-note beat timings. **(C)** Red circles and line represent the change of inter-onset interval (IOI) in the tap signal. The blue dashed line represents the change of beat intervals. The slope of linear regression function of the tap IOI was calculated (pink line). The participant's beat production was categorized as slowing down when the slope was positive whereas categorized as speeding up when negative.

We created and repeated a series of notes that consisted of one quarter note, two eighth notes, one dotted-quarter note, and one eighth note (Figure 7A and Supplementary Material). The positions of quarter-note beats underlying this rhythmic pattern were shown as arrows in Figure 7A. A tone sequence consisted of 1 pure tone and 27 woodblock tones corresponding to 21 quarter-note beats (Figure 7B). We used the following temporal-change rule to make the inter-beat interval (IBI) slowing down or speeding up.

(5) $$\{\begin{array}{c} IBI_{i+1}=IBI_{i}+d\;(\text{slower})\\ IBI_{i+1}=IBI_{i}-d\;(\text{faster}) \end{array}$$

Note that, the ISI was changed in Equation 4 whereas the IBI was changed in Equation 5: For example, if *d* = 10 and the tempo was slowing down, the first four IBIs were 500, 510, 520, and 530 in ms. In this case, the ISI between the woodblock tones were set as follows: 500 ms (quarter note), 510/2 = 255 ms (eighth note), 510/2 = 255 ms (eighth note), 520 + 530/2 = 785 ms (dotted-quarter note), and 530/2 = 265 ms (eighth note). The parameter *d* was started from 20 ms to make it the same as in the BIT. The *d* was then manipulated according to the stair-case paradigm (see the section called “stair-case paradigm” below for the detail). The pure tone was played 1 s before the woodblock tones, the same as was done in the BST and BIT.

There were two parts in the BFIT. First, the participants had to find the underlying quarter-note beat and discriminate its temporal-change pattern (slower/faster) perceptually via the keyboard (Figure 2A). Second, they had to find and produce the quarter-note beat by tapping on the drum pad (Figure 2B). In the perception part, the participants had to decide which temporal-change pattern they were hearing by pressing either the *Z* (slower) or *X* (faster) buttons, and pressed the *Enter* afterwards. Again, the participants were not allowed to move other parts of their body except for the fingers used to press the buttons in order to measure the pure perception response.

In the production part, we detected the tap onsets during tapping the beats with the rhythmic pattern (red circles in Figure 7B). We discarded the first-two and the last-two tap onsets to eliminate the start-up and wind-down effects. We calculated the slope of linear regression function of the tap IOIs (Figure 7C). We categorized the beat production as slowing down when the slope was positive, while as speeding up otherwise. If the participant could find the quarter-note beat and adjust to the temporal change correctly, the participant's response should match with the stimulus pattern.

### Stair-case paradigm

**Figure 8:**
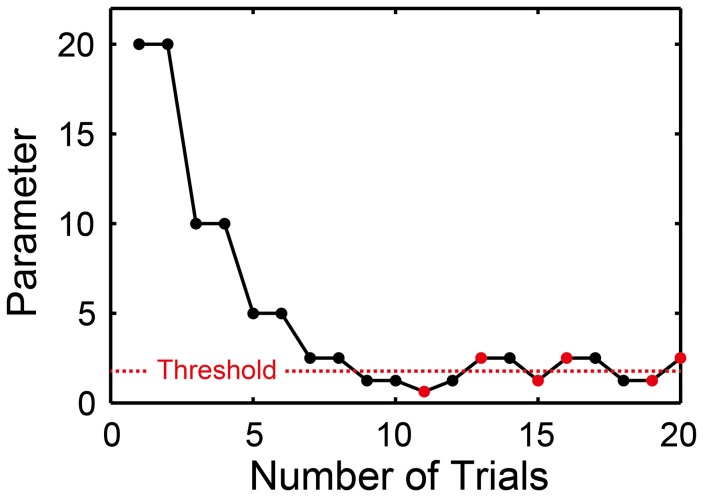
**Two-down one-up stair-case method used in beat saliency test (BST), beat interval test (BIT), and beat finding and interval test (BFIT)**. The parameter was halved when the pattern of the stimulus matched with the participant's response twice consecutively, while doubled otherwise. Every time the direction of parameter change reversed from down to up or from up to down, the parameter at which this occurred was recorded as turnaround point (marked by red). We calculated the average across the six turnaround points and defined it as a threshold (horizontal dashed line).

In order to measure the thresholds to perceive and produce the meter/beat, we used an adaptive two-alternative forced-choice discrimination paradigm (Levitt, 1971) for the BST, BIT, and BFIT using the following starting parameters: The relative-intensity difference between the accented and unaccented tones was started from 20 dB in the BST, and the change of ISI was started from 20 ms in the BIT and BFIT (i.e., the parameter *d* in Equations 4 and 5 was started from 20, see also Figure 8).

In each trial, one of the two patterns (i.e., duple or triple in the BST, slower or faster in the BIT and BFIT) was provided randomly to the participants. We categorized the participant's response by the keyboard in the perception part and by the tap signal in the production part (based on the auto-correlation function or the slope of linear regression line, see also Figure 2). The current parameter was halved (e.g., from 20 to 10, 5, and 2.5…) when the pattern of the stimulus matched with the participant's response twice consecutively, and doubled otherwise. That is, we performed two-down one-up stair-case paradigm (Figure 8). Every time the direction of parameter change reversed from down to up or from up to down, the parameter at which this occurred was recorded as turnaround point. One run of the task continued until the six turnaround points were collected. We calculated the average across the six turnaround points and defined it as a threshold (horizontal dashed line in Figure 8).

We performed two runs to measure perception and production thresholds for each of the BST, BIT, and BFIT (i.e., 6 runs in total). The participants practiced both of the two patterns (i.e., duple/triple or slower/faster) at a starting parameter (i.e., 20 dB or 20 ms) before running the threshold measurement. We asked them to close their eyes to prevent the effect of visual feedback in both perception and production parts during the measurements. The six runs measuring the thresholds were performed after finishing the MTT. Thus, the order of the task was; hearing test—MTT—six stair-case runs. The order of the six runs was randomized across the participants.

### Statistics

There were 9 behavioral variables in the H-BAT; 3 synchronization indices (*SIs*) and 6 thresholds. The *SI*_ARV_ and *SI*_ENT_ fulfilled the criteria of normal distribution (The Kolmogorov-Smirnov test, *SI*_ARV_, *D*_30_ = 0.11, *p* = 0.20; *SI*_ENT_, *D*_30_ = 0.16, *p* = 0.06) while the *SI*_LRV_did not (*D*_30_ = 0.21, *p* < 0.01). Four out of six raw thresholds did not fulfill the criteria of normal distribution (*D*_30_ = 0.13 ~ 0.24, *p* = 0.20 ~ 0.0002) whereas all the thresholds with logarithmic scale of base two did (*D*_30_ = 0.08 ~ 0.16, *p* = 0.20 ~ 0.06). Thus, we used logarithmic-scaled thresholds in the following statistical tests. The years of duration of musical training fulfilled the criteria of normal distribution (*D*_30_ = 0.09, *p* = 0.20) while the estimated hours of practice did not (*D*_30_ = 0.18, *p* < 0.05).

We performed paired *t*-test to examine the difference between the perception and production thresholds with a significance level at *p* < 0.05. We also calculated Cohen's *d* as a measure of the effect size. To examine the relationship among the subtests, we calculated Pearson's correlation coefficients across the 9 behavioral variables. We used a significance level at *p* < 0.05 for this correlation analysis. To investigate the effect of musical training, the 9 behavioral variables were also correlated with the years of duration of musical training and the estimated amount of musical training using Spearman's rank correlation coefficient. We used Bonferroni correction in order to compensate for the increased probability of finding significant results for the correlations with duration and amount of musical training (i.e., *p* < 0.05/9 = 0.0056). We also calculated the testing duration of each sub-task using the time-stamp information of the recorded data files. The testing duration was averaged across the participants.

## Results

### Testing duration

**Figure 9:**
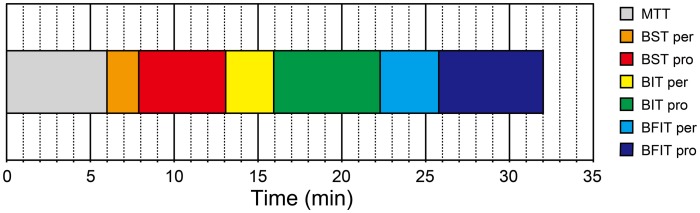
**Testing duration of the Harvard Beat Assessment Test (H-BAT)**. MTT, music tapping test; BST per, perception part of beat saliency test; BST pro, production part of beat saliency test; BIT per, perception part of beat interval test; BIT pro, production part of beat interval test; BFIT per, perception part of beat finding and interval test; BFIT pro, production part of beat finding and interval test.

The mean testing duration of H-BAT was 32.01 ± 2.26 min in total (Figure 9); MTT = 5.99 ± 0.18 min, BSTper = 1.90 ± 0.42 min, BSTpro = 5.18 ± 0.90 min, BITper = 2.86 ± 1.07 min, BITpro = 6.35 ± 0.90 min, BFITper = 3.50 ± 1.06 min, BFITpro = 6.23 ± 1.49 min. The hearing test prior to the H-BAT took about 1 min.

### H-BAT scores

**Figure 10:**
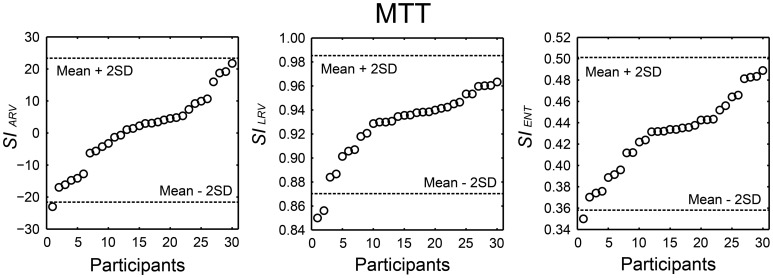
**Distribution of the synchronization indices (*SI*s) calculated from angle of resultant vector (*SI*_ARV_), length of resultant vector (*SI*_LRV_), and entropy of relative phase distribution (*SI*_ENT_) obtained from 30 participants performing the music tapping test (MTT)**. Horizontal dashed lines indicate cut-off scores (mean ± 2 *SD*). The participant's order is sorted from smallest to largest.

We summarized the H-BAT scores from the 30 participants in Table 1. It shows mean, standard deviation (SD), mean ± 2*SD* values, minimum, maximum, range, percentiles, skewness, and kurtosis. The thresholds were shown in both raw and logarithmic scales. The distributions of the synchronization indices (*SIs*) are shown in Figure 10 with cut-off lines of mean ± 2*SD*.

**Table 1:** **Scores in the Harvard Beat Assessment Test (H-BAT) (*n* = 30)**.

**Task**	**MTT**			**BST (dB)**		**BIT (ms)**		**BFIT (ms)**		**BST (Log2dB)**		**BIT (Log2ms)**		**BFIT (Log2ms)**	
	**SI_ARV_**	**SI_LRV_**	**SI_ENT_**	***per***	***pro***	***per***	***pro***	***per***	***pro***	***per***	***pro***	***per***	***pro***	***per***	***pro***
Mean	0.89	0.928	0.430	2.16	2.21	1.83	0.48	1.18	0.76	0.88	0.80	0.33	−1.28	−0.13	−0.78
*SD*	11.24	0.029	0.036	1.27	1.79	1.48	0.25	0.95	0.57	0.84	1.04	1.41	0.90	1.09	1.12
Mean − 2*SD*	−21.60	0.870	0.358	−0.38	−1.36	−1.12	−0.01	−0.72	−0.37	−0.80	−1.28	−2.48	−3.09	−2.31	−3.03
Mean + 2*SD*	23.38	0.985	0.501	4.70	5.79	4.79	0.97	3.09	1.90	2.56	2.88	3.14	0.52	2.04	1.46
Min	−22.99	0.850	0.350	0.55	0.27	0.16	0.07	0.12	0.12	−0.87	−1.87	−2.68	−3.94	−3.09	−3.09
Max	21.76	0.963	0.489	5.94	9.58	5.83	1.08	4.38	2.50	2.57	3.26	2.54	0.11	2.13	1.32
Range	44.75	0.113	0.139	5.39	9.31	5.68	1.02	4.26	2.38	3.44	5.13	5.22	4.05	5.22	4.42
Percentile															
5	−19.67	0.853	0.361	0.59	0.39	0.17	0.09	0.20	0.14	−0.76	−1.42	−2.58	−3.47	−2.42	−2.86
10	−16.00	0.884	0.374	0.73	0.63	0.23	0.14	0.35	0.19	−0.45	−0.68	−2.18	−2.82	−1.51	−2.37
25	−5.82	0.915	0.408	1.24	1.22	0.74	0.34	0.58	0.35	0.31	0.29	−0.44	−1.57	−0.79	−1.51
50	2.63	0.936	0.434	1.95	1.88	1.39	0.42	0.91	0.61	0.96	0.91	0.48	−1.24	−0.13	−0.71
75	7.83	0.945	0.453	2.53	2.50	2.73	0.63	1.48	1.13	1.34	1.32	1.45	−0.68	0.57	0.18
90	18.47	0.960	0.482	4.25	3.72	4.06	0.90	2.18	1.55	2.08	1.89	2.02	−0.16	1.13	0.63
95	20.34	0.962	0.486	5.59	7.52	5.37	1.00	4.03	2.16	2.48	2.87	2.42	0.00	2.01	1.09
Skewness	−0.20	−1.292	−0.359	1.44	2.75	1.11	0.62	2.03	1.29	−0.19	−0.32	−0.58	−1.08	−0.30	−0.17
Kurtosis	−0.31	1.425	−0.312	2.45	9.83	0.72	0.10	4.63	1.68	−0.03	1.16	−0.27	1.67	1.03	−0.69

H-BAT consists of four subtests; Music Tapping Test (MTT), Beat Saliency Test (BST), Beat Interval Test (BIT), and Beat Finding and Interval Test (BFIT). The MTT has three Synchronization Indices (SIs) calculated from angle of resultant vector (SI_ARV_), length of resultant vector (SI_LRV_), and entropy of relative phase distribution (SI_ENT_). Note that SI_ARV_ and SI_LRV_ are expressed in degrees. The BST, BIT, and BFIT assess a perception (per) and production (pro) threshold each.

**Figure 11:**
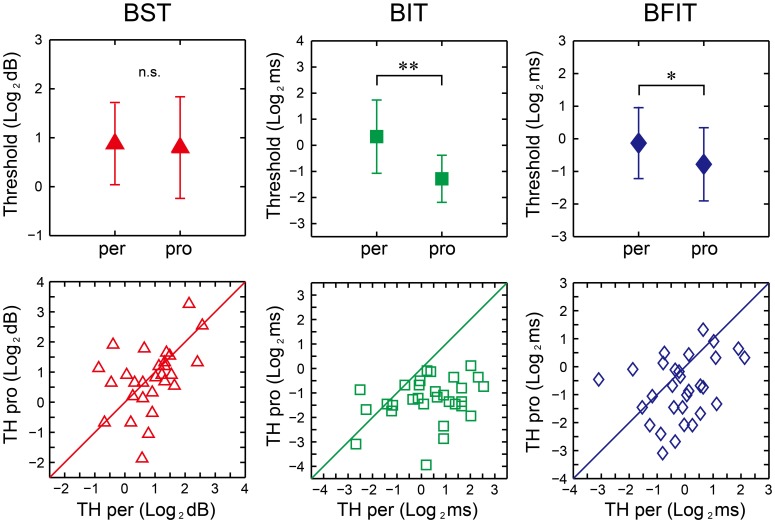
**Mean (upper) and distribution (lower) of the thresholds across 30 participants in beat saliency test (BST), beat interval test (BIT), and beat finding and interval test (BFIT)**. The error bars indicate the standard deviation (*SD*) across the participants. Diagonal line represents *y* = *x*. ^*, **^significant at *p* < 0.05 and *p* < 0.01, respectively. per, perception; pro, production; TH, threshold.

There were no significant differences between the perception and production thresholds in the BST [*t*_(29)_ = 0.44, *p* = 0.66, *d* = 0.09], whereas the production thresholds were significantly lower than the perception one in the BIT and BFIT [*t*_(29)_ = 6.07, *p* < 0.0001, *d* = 1.37; *t*_(29)_ = 2.74, *p* < 0.05, *d* = 0.59, respectively; see upper part in Figure 11].

**Figure 12:**
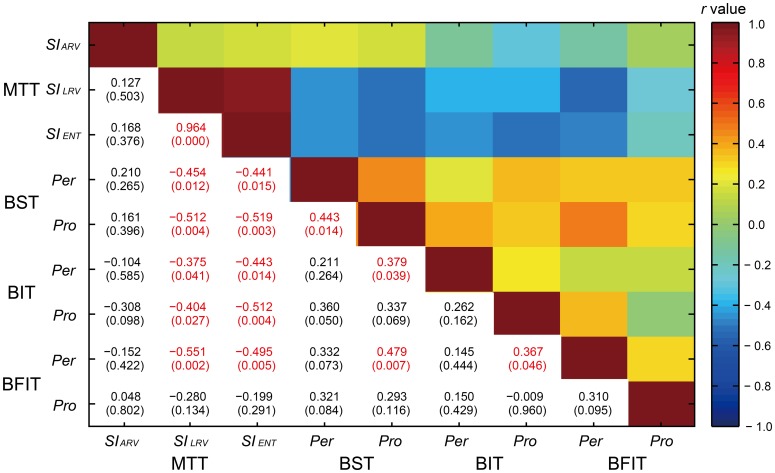
**A correlation matrix across the 9 behavioral variables in the Harvard Beat Assessment Test (H-BAT) using Pearson's correlation coefficients (*r*)**. The values in parenthesis indicate *p*-values (red text, significant at *p* < 0.05). MTT, music tapping test; BST, beat saliency test; BIT, beat interval test; BFIT, beat finding and interval test; pro, production part; Per, perception part; *SI*_ARV_, synchronization index calculated from angle of resultant vector; *SI*_LRV_, synchronization index calculated from length of resultant vector; *SI*_ENT_, synchronization index calculated from entropy of relative-phase distribution.

The correlations across the 9 behavioral variables are shown in Figure 12. There was a high correlation between the *SI*_LRV_ and *SI*_ENT_ [*r*_(30)_ = 0.964, *p* < 0.0001]. The measures of synchronization consistency (*SI*_LRV_ and *SI*_ENT_) were negatively correlated with the perception/production thresholds (see light blue colors in Figure 12); the higher the *SI*_LRV_ and *SI*_ENT_, the lower the thresholds [*r*_(30)_ = −0.551 ~ −0.199, *p* = 0.002 ~ 0.291, 10 out of 12 *p*-values reached at the level of 0.05].

We found significant correlation between the perception and production thresholds in the BST [*r*_(30)_ = 0.443, *p* < 0.05], while no significant correlation was found either in the BIT or BFIT [*r*_(30)_ = 0.262, *p* = 0.162; *r*_(30)_ = 0.310, *p* = 0.095, respectively; see lower part in Figures 11, 12]. There were some other significant correlations among the thresholds; the lower the production threshold in the BST, the lower the perception thresholds in the BIT and BFIT [*r*_(30)_ = 0.379, *p* < 0.05; *r*_(30)_ = 0.479, *p* < 0.01, respectively]; the lower the production threshold in the BIT, the lower the perception threshold in the BFIT [*r*_(30)_ = 0.367, *p* < 0.05].

The duration of musical training was significantly correlated with the *SI*_LRV_ and *SI*_ENT_ (*SI*_LRV_, ρ_30_ = 0.554, *p* < 0.0056; *SI*_ENT_, ρ_30_ = 0.540, *p* < 0.0056). The estimated hours of musical training was also significantly correlated with the *SI*_LRV_ and *SI*_ENT_ (*SI*_LRV_, ρ_30_ = 0.623, *p* < 0.0056; *SI*_ENT_, ρ_30_ = 0.617, *p* < 0.0056): The more musical training, the higher the degree of synchronization with the musical beat. The estimated hours of musical training was also correlated with the production threshold in the BIT (ρ_30_ = −0.511, *p* < 0.0056): The more musical training, the lower the production threshold in the BIT. The other correlations with the duration and amount of musical training did not reach the significant level (ρ_30_ = −0.479 ~ −0.229, *p* = 0.007 ~ 0.224).

## Discussion

### Testing duration

The H-BAT took only 32 min in total (Figure 9). The whole experimental duration would be less than 1 h accounting for additional procedures such as task instructions, filling out questionnaires, device setup, and etc. This is shorter than the MBEA (1.5 h, Peretz et al., 2003) and the BAASTA (2–3 h, Farrugia et al., 2012). Thus, the H-BAT can be performed in a relatively short period of time. A major challenge in the study of beat-deafness is that it is difficult to find many cases of a presumed rare disorder such as beat-deafness (Phillips-Silver et al., 2011). A short-testing duration, such as the duration of the H-BAT, would be helpful in future studies if one wants to test a large sample of subjects in an efficient way.

### Synchronization indices

We developed the MTT and the *SI*s to quantify an individual's ability to synchronize the tapping movement with the musical beat. The data set from 30 individuals showed that *SI*s varied widely across individuals (Figure 10). The cut-off scores of mean – 2SD and the 5th percentiles of the *SI*s are thought to be useful objective measures to identify individuals who might be more or less sensitive to perceive or synchronize with a beat in future studies.

In this study, 18 out of 30 participants showed a positive value of *SI*_ARV_ and the mean across the participants was about zero (see Table 1). The lack of overall negative mean asynchrony (e.g., Aschersleben, 2002) when tapping to the music is consistent with previous studies (Large, 2000; Snyder and Krumhansl, 2001). The *SI*_ARV_ and *SI*_ENT_ fulfilled the prerequisite for normal distribution, while the *SI*_LRV_ did not. This could be because the *SI*_LRV_ tended to be under-estimated when the relative phase was distributed around 180 degree (e.g., Figure 4). Although the experimenter instructed the participants not to tap the eighth-note or syncopated-fourth-note beats, some of them seemed to tap around 180 degree accidentally. This could lead to small *SI*_LRV_values in some of the participants resulting in the asymmetric distribution (see middle part of Figure 10). In contrast, the *SI*_ENT_ is more robust to the bimodal phase distribution around in- and anti-phases since it is the logarithm of the probability density function (Figure 4 and Equations 2 and 3). This could be the reason why the *SI*_ENT_ was distributed more normally than the *SI*_LRV_. During playing music, playing of the eighth- and syncopated-fourth-note beats can be regarded as musically natural depending on the hierarchical structure or subdivision of beats. Therefore, we think that the *SI*_LRV_ might be problematic if it under-estimates the degree of synchronization because of the distribution around 180 degree (i.e., anti-phase). It might be better to use the *SI*_ENT_ as a measure of synchronization consistency compared with the *SI*_LRV_ for the study of beat-deafness.

### Perception and production threshods

The BST, BIT, and BFIT were developed to assess basic abilities needed to process the beat. The BST assessed the thresholds to perceive/produce the meter based on the beat saliency, while the BIT and BFIT assessed the thresholds to perceive/produce the beat-interval change in the isochronous and non-isochronous sequences.

The mean perception and production thresholds in the BST were 2.16 and 2.21 dB, respectively (Table 1). It ranged from 0.55 to 5.94 dB in the perception part and from 0.27 to 9.58 dB in the production part; there were individual differences in the thresholds. The values are comparable with the previous study by Chen et al. (2006) who used intensity accents consisting of 0-, 1-, 2-, 6-, or 10-dB attenuation between unaccented and accented tones. They reported that this ranged from a level at which participants could not detect the accents, to a level where they were able to notice it sufficiently (Chen et al., 2006). This is consistent with the range of the threshold in this study.

The raw thresholds values were overall similar between the BIT and BFIT. This is consistent with previous studies that showed similar tapping adaptations in response to temporal perturbations in both isochronous and non-isochronous metrical sequences (Large et al., 2002; Repp et al., 2008). However, it is important to note that the thresholds were not correlated between the BIT and BFIT (the perception thresholds were not correlated between the two tasks, nor were the production thresholds, see Figure 12). These results indicate that the individual performance was not distributed in the same way. Thus, it is important to perform both BIT and BFIT to investigate individual differences.

The mean perception thresholds in the BIT and BFIT were 1.83 ms (0.366% initial ISI) and 1.18 ms (0.236% initial IBI), respectively. The accumulated amount of interval change across 20 ISIs were 36.60-ms (7.32% initial ISI) and 23.60-ms (4.72% initial IBI), respectively. These perceptual thresholds in this study were lower than what is reported in a previous study using the continuous tempo change in 500-ms ISI sequence (Madison, 2004), which showed the threshold was about 10 ms (2% initial ISI)/interval across 9 ISIs [i.e., about 90 ms (18% initial ISI) in total]. This might be attributed to the differences in the experimental paradigm and the musical experience of the participants: (1) the number of tones and total duration of sequence were different compared to the Madison's study, (2) the present study asked the participants to discriminate slower/faster in relatively long tone sequence while Madison's study asked whether or not tempo irregularity existed in relatively short tone sequence, and (3) the participants in this study had on average a longer duration of musical training (11.8 years) compared to the Madison's study (6.7 years on average). Actually, another study by Kuhn (1974) showed that musicians were able to respond to a gradual 6-BPM change over 6 s in 400-ms ISI or 150-BPM metronome setting (i.e., 1.08-ms or 0.27%-ISI change per interval). The values are comparable with this study. The experimental paradigm was also somewhat similar with Kuhn's study (1974); Kuhn asked the participants to discriminate whether the metronome was getting slower, faster, or revealed no change.

We found that the production thresholds were overall significantly lower than the perception ones in the BIT and BFIT [0.48 ms (0.096% initial ISI) and 0.76 ms (0.152% initial IBI)], respectively. The values correspond to accumulated amount of 9.60-ms (1.92% initial ISI) and 15.20-ms (3.04% initial IBI) changes across 20 intervals. That is, the participants in this study were able to adapt to the direction of temporal change by their tapping even at the level in which they could not discriminate in the perception tasks. These results are consistent with previous findings that the tapping adaptation occurred well below the explicit perceptual detection threshold (Repp, 2000, 2001; Repp and Keller, 2004; Repp, 2005). One of the reasons for this could be that the participants could use additional interval information provided by the motor system. This additional motor information could also interact with short-term memory (Brown and Palmer, 2012), allowing participants to remember intervals over longer time scales. In other words, it might be possible that the act of tapping enabled the comparison of more widely spaced intervals (e.g., the first interval and the last of a sequence, while pure perception might be more limited to more local comparisons). The finding of better performance in the production than the perception is also consistent with tone-deaf studies by Loui et al. (2008, 2009): the individuals, who cannot consciously perceive pitch directions, can paradoxically reproduce pitch intervals in correct directions. Nevertheless, our results indicate that the perception and production parts in the BIT and BFIT were processed in different ways.

### Relationship among the subtests

We found that the measures of synchronization consistency (the *SI*_LRV_ and *SI*_ENT_) in the MTT were negatively correlated with the perception/production thresholds in the BST, BIT, and BFIT (Figure 12); the individual, who showed less degree of synchronization, had a tendency to have higher perception and production thresholds. The results support our assumption that the abilities to perform the BST, BIT and BFIT are sharing the common processes with those during synchronizing to the musical beat.

We found significant correlation between the perception and production thresholds in the BST, showing that the individual, who had higher perception threshold, also had higher production threshold. On the other hand, there was no significant correlation between the perception and production thresholds in the BIT and BFIT, suggesting that the thresholds were less tightly coupled. When we looked at the individual plot, some had higher (worse) perception thresholds with normal or lower (better) production thresholds than the overall group, and some had higher (worse) production thresholds with normal or lower (better) perception thresholds (see lower part in Figure 11). These results suggest that a dissociation could exist between perception and production.

It is interesting that the production threshold of the BST correlated with the perception thresholds in the BIT and BFIT. This might be because the production part of BST included not only the meter-discrimination but also a time-interval control process to tap in synchrony with the tones. The correlation between the production threshold in the BIT and the perception threshold in the BFIT might reflect a covariate that related to the adaptation of internal timekeeper, but the result is difficult to interpret considering the fact that the other correlations between the BIT and BFIT were not significant (Figure 12). It is also interesting (but difficult to interpret) that we could not observe significant correlations across the production tasks since one could expect a covariate related to the motor implementation of tapping. One could also assume that basic duration and volume detection abilities outside of a beat content act as covariates across the tasks in the H-BAT. Nevertheless, more studies are needed to further explore the relationship among the subtests in the H-BAT.

### Effect of musical training

The duration and amount of musical training were significantly correlated with the *SI*_LRV_ and *SI*_ENT_ in this study. That is, the individuals who had more musical training showed higher degree of synchronization with the musical beat. Thus, the MTT and the *SI*s could be sensitive to the effect of musical training. Moreover, we found the significant correlation of the estimated hours of musical training only with the production threshold in the BIT, whereas the other correlations did not reach significance. If distinct neural circuits are involved in performing the BST, BIT, and BFIT, then one explanation is that musical training elicits more plasticity in certain neural circuits compared to others.

### Implications for beat-deaf studies

In the previous study by Phillips-Silver et al. (2011), the beat-deaf individual (called Mathieu) could not synchronize with the beat of music but also failed to discriminate duple and triple meter. In addition, the beat-deaf individual could not adapt his tapping to tempo change in music while he was able to adapt to isochronous tone sequences (i.e., Mathieu could synchronize with a metronome but not with music) (Phillips-Silver et al., 2011). It would be interesting if one could perform the H-BAT on Mathieu. Based on the report by Phillips-Silver et al. (2011), he might show lower *SI*_ENT_ (*SI*_LRV_) in the MTT and higher thresholds in the BST. He might also show relatively normal thresholds in the BIT and/or BFIT. In the other beat-deaf study by Sowinski and Dalla Bella (2013), two individuals failed to synchronize with the music beat while they were able to discriminate tone-interval patterns. Again, if tested on the H-BAT, they might show lower *SI*_ENT_ (*SI*_LRV_) in the MTT while having normal thresholds in the BIT and BFIT. The H-BAT might provide further insights into the beat processing in humans in terms of determining which ability is intact (or impaired) in beat-deaf individuals.

## Conclusions

In this paper, we proposed the H-BAT, a battery of tests to assess beat perception and production abilities. We showed that the H-BAT can be performed within a reasonable period of time. The *SI*s in the MTT might be objective measures to identify individuals who deviate from group mean performance, while the thresholds in the BST, BIT, and BFIT might be useful to investigate the dissociation between perception and production. It is worth mentioning that previous neuroimaging studies were taking into consideration when we developed the BST, BIT, and BFIT. That is, the thresholds may be related to distinct neural circuits in auditory-premotor (Chen et al., 2006; Grahn and Rowe, 2009), striatal-thalamo-cortical and olivocerebellar systems (Grahn and Rowe, 2009; Grube et al., 2010; Teki et al., 2011a,b), and/or prefrontal networks (Chen et al., 2008b). Future studies using the H-BAT together with neuroimaging techniques will help to reveal underlying neural correlates of beat-processing mechanisms in the human brain.

### Conflict of interest statement

The authors declare that the research was conducted in the absence of any commercial or financial relationships that could be construed as a potential conflict of interest.

## Supplementary material

The Supplementary Material for this article can be found online at: http://www.frontiersin.org/journal/10.3389/fnhum.2013.00771/abstract (the sound stimuli in the Harvard Beat Assessment Test. A file name in Music Tapping Task (MTT); e.g., “ACL_120.mp3,” A Chorus Line at 120 beat per minute (HSG, Hurts So Good by J. Mellencamp; TJU, Tuxedo Junction by Glenn Miller). A file name in Beat Saliency Task (BST); e.g., “BST_500_D(T)_20_02.mp3,” it has 500-ms inter-stimulus interval (ISI), Duple (Triple) meter, initial parameter of 20 dB in the stair-case method (see Figure 8), and it is 2nd level in the set of stimuli [1st level is 20-dB difference between the accented and unaccented tones, 2nd level is 20/2 = 10 dB, and 3rd level is 20/2/2 = 5 dB…]. A file name in beat interval task (BIT) and beat finding and interval task (BFIT); e.g., “BIT_500_F(S)_20_03.mp3,” it is beat interval task, it has 500-ms ISI, the tempo becomes Faster (Slower), the initial parameter is 20 ms in the stair-case method, and it is 3rd level in the set of stimuli [1st level is tempo change of 20 ms per interval, 2nd level is 20/2 = 10 ms, and 3rd level is 20/2/2 = 5 msec…).

## References

[B1] Aschersleben G. (2002). Temporal control of movements in sensorimotor synchronization. Brain Cogn. 48, 66–79 10.1006/brcg.2001.1304 11812033

[B2] Brown R. M. Chen J. L. Hollinger A. Penhune V. B. Palmer C. Zatorre R. J. (2013). Repetition suppression in auditory-motor regions to pitch and temporal structure in music. J. Cogn. Neurosci. 25, 313–328 10.1162/jocn_a_00322 23163413

[B3] Brown R. M. Palmer C. (2012). Auditory-motor learning influences auditory memory for music. Mem. Cognit. 40, 567–578 10.3758/s13421-011-0177-x 22271265

[B4] Buhusi C. V. Meck W. H. (2005). What makes us tick? Functional and neural mechanisms of interval timing. Nat. Rev. Neurosci. 6, 755–765 10.1038/nrn1764 16163383

[B5] Chen J. L. Penhune V. B. Zatorre R. J. (2008a). Listening to musical rhythms recruits motor regions of the brain. Cereb. Cortex 18, 2844–2854 10.1093/cercor/bhn042 18388350

[B6] Chen J. L. Penhune V. B. Zatorre R. J. (2008b). Moving on time: brain network for auditory-motor synchronization is modulated by rhythm complexity and musical training. J. Cogn. Neurosci. 20, 226–239 10.1162/jocn.2008.20018 18275331

[B7] Chen J. L. Zatorre R. J. Penhune V. B. (2006). Interactions between auditory and dorsal premotor cortex during synchronization to musical rhythms. Neuroimage 32, 1771–1781 10.1016/j.neuroimage.2006.04.207 16777432

[B8] Cooper G. Meyer L. B. (1960). The Rhythmic Structure of Music. Chicago, IL: The University of Chicago Press

[B9] Drake C. Jones M. R. Baruch C. (2000). The development of rhythmic attending in auditory sequences: attunement, referent period, focal attending. Cognition 77, 251–288 10.1016/S0010-0277(00)00106-2 11018511

[B10] Ellis R. J. Jones M. R. (2009). The role of accent salience and joint accent structure in meter perception. J. Exp. Psychol. Hum. Percept. Perform. 35, 264–280 10.1037/a0013482 19170487

[B11] Farrugia N. Benoit C. E. Harding E. Kotz S. A. Dalla Bella S. (2012). BAASTA: battery for the assessment of auditory sensorimotor and timing abilities, in The Joint Conference: 12th Biennial International Conference on Music Perception and Cognition and 8th Triennial Conference of the European Society for the Cognitive Sciences of Music (ICMPC-ESCOM 2012), eds Cambouropoulos E. Tsougras P. Mavromatis P. Pastiadis K. (Thessaloniki).

[B12] Foxton J. M. Nandy R. K. Griffiths T. D. (2006). Rhythm deficits in ‘tone deafness’. Brain Cogn. 62, 24–29 10.1016/j.bandc.2006.03.005 16684584

[B13] Fujii S. Hirashima M. Kudo K. Ohtsuki T. Nakamura Y. Oda S. (2011). Synchronization error of drum kit playing with a metronome at different tempi by professional drummer. Music Percept. 28, 491–503 10.1525/mp.2011.28.5.491

[B14] Fujii S. Kudo K. Ohtsuki T. Oda S. (2010). Intrinsic constraint of asymmetry acting as a control parameter on rapid, rhythmic bimanual coordination: a study of professional drummers and nondrummers. J. Neurophysiol. 104, 2178–2186 10.1152/jn.00882.2009 20702735 PMC2957461

[B15] Fujii S. Oda S. (2009). Effect of stick use on rapid unimanual tapping in drummers. Percept. Mot. Skills 108, 962–970 10.2466/pms.108.3.962-970 19725329

[B16] Grahn J. A. (2012). Neural mechanisms of rhythm perception: current findings and future perspectives. Top Cogn. Sci. 4, 585–606 10.1111/j.1756-8765.2012.01213.x 22811317

[B17] Grahn J. A. Rowe J. B. (2009). Feeling the beat: premotor and striatal interactions in musicians and nonmusicians during beat perception. J. Neurosci. 29, 7540–7548 10.1523/JNEUROSCI.2018-08.2009 19515922 PMC2702750

[B18] Grahn J. A. Shuit D. (2012). Individual differences in rhythmic ability: behavioral and neuroimaging investigations. Psychomusicology 22, 105–121 10.1037/a0031188

[B19] Griffiths T. D. (2008). Sensory systems: auditory action streams? Curr. Biol. 18, R387–R388 10.1016/j.cub.2008.03.007 18460320

[B20] Grube M. Cooper F. E. Chinnery P. F. Griffiths T. D. (2010). Dissociation of duration-based and beat-based auditory timing in cerebellar degeneration. Proc. Natl. Acad. Sci. U.S.A. 107, 11597–11601 10.1073/pnas.0910473107 20534501 PMC2895141

[B21] Hickok G. Poeppel D. (2004). Dorsal and ventral streams: a framework for understanding aspects of the functional anatomy of language. Cognition 92, 67–99 10.1016/j.cognition.2003.10.011 15037127

[B22] Hyde K. L. Lerch J. P. Zatorre R. J. Griffiths T. D. Evans A. C. Peretz I. (2007). Cortical thickness in congenital amusia: when less is better than more. J. Neurosci. 27, 13028–13032 10.1523/JNEUROSCI.3039-07.2007 18032676 PMC6673307

[B23] Hyde K. L. Peretz I. (2004). Brains that are out of tune but in time. Psychol. Sci. 15, 356–360 10.1111/j.0956-7976.2004.00683.x 15102148

[B24] Hyde K. L. Zatorre R. J. Peretz I. (2011). Functional MRI evidence of an abnormal neural network for pitch processing in congenital amusia. Cereb. Cortex 21, 292–299 10.1093/cercor/bhq094 20494966

[B25] Iversen J. R. Patel A. D. (2008). The beat alignment test (BAT): Surveying beat processing abilities in the general population, in The 10th International Conference on Music Perception, and Cognition (ICMPC 10), eds Miyazaki M. Hiraga Y. Adachi M. Nakajima Y. Tsuzaki M. (Sapporo).

[B26] Koelsch S. (2012). Brain and Music. Oxford: John Wiley and Sons Ltd

[B27] Kuhn T. L. (1974). Discrimination of modulated beat tempo by professional musicians. J. Res. Music Edu. 22, 270–277 10.2307/3344764

[B28] Large E. W. (2000). On synchronizing movements to music. Hum. Mov. Sci. 19, 527–566 10.1016/S0167-9457(00)00026-9

[B29] Large E. W. (2008). Resonating to musical rhythm: theory and experiment, in Psychology of Time, ed Grondin S. (Bingley: Emerald), 189–231

[B30] Large E. W. Fink P. Kelso J. A. (2002). Tracking simple and complex sequences. Psychol. Res. 66, 3–17 10.1007/s004260100069 11963276

[B31] Lartillot O. Eerola T. Toiviainen P. Fornari J. (2008a). Multi-feature modeling of pulse clarity: design, validation, and optimization, in The 11th International Conference on Digital Audio Effects, (Espoo).

[B32] Lartillot O. Toiviainen P. Eerola T. (2008b). A Matlab toolbox for music information retrieval, in Data Analysis, Machine Learning and Applications, (Berlin; Heidelberg: Springer), 261–268 10.1007/978-3-540-78246-9_31

[B33] Lartillot O. Toiviainen P. (2007). A Matlab toolbox for musical feature extraction from audio, in The 10th Conference on Digital Audio Effects, (Bordeaux).

[B34] Law L. N. Zentner M. (2012). Assessing musical abilities objectively: construction and validation of the profile of music perception skills. PLoS ONE 7:e52508 10.1371/journal.pone.0052508 23285071 PMC3532219

[B35] Lerdahl F. Jackendoff R. (1983). A Generative Theory of Tonal Music. Cambridge, MA: MIT Press

[B36] Levitt H. (1971). Transformed up-down methods in psychoacoustics. J. Acoust. Soc. Am. 49, 467–477 10.1121/1.1912375 5541744

[B37] Loui P. Alsop D. Schlaug G. (2009). Tone deafness: a new disconnection syndrome? J. Neurosci. 29, 10215–10220 10.1523/JNEUROSCI.1701-09.2009 19692596 PMC2747525

[B38] Loui P. Guenther F. H. Mathys C. Schlaug G. (2008). Action-perception mismatch in tone-deafness. Curr. Biol. 18, R331–R332 10.1016/j.cub.2008.02.045 18430629 PMC2791531

[B39] Madison G. (2004). Detection of linear temporal drift in sound sequences: empirical data and modelling principles. Acta Psychol. (Amst) 117, 95–118 10.1016/j.actpsy.2004.05.004 15288231

[B40] Mandell J. Schulze K. Schlaug G. (2007). Congenital amusia: an auditory-motor feedback disorder? Restor. Neurol. Neurosci. 25, 323–334 17943009

[B41] Mase M. Faes L. Antolini R. Scaglione M. Ravelli F. (2005). Quantification of synchronization during atrial fibrillation by Shannon entropy: validation in patients and computer model of atrial arrhythmias. Physiol. Meas. 26, 911–923 10.1088/0967-3334/26/6/003 16311441

[B42] Mates J. (1994). A model of synchronization of motor acts to a stimulus sequence. I. Timing and error corrections. Biol. Cybern. 70, 463–473 10.1007/BF00203239 8186306

[B43] Mathys C. Loui P. Zheng X. Schlaug G. (2010). Non-invasive brain stimulation applied to Heschl's gyrus modulates pitch discrimination. Front. Psychol. 1:193 10.3389/fpsyg.2010.00193 21286253 PMC3028589

[B44] Oldfield R. C. (1971). The assessment and analysis of handedness: the Edinburgh inventory. Neuropsychologia 9, 97–113 10.1016/0028-3932(71)90067-4 5146491

[B45] Peretz I. Champod A. S. Hyde K. (2003). Varieties of musical disorders. the montreal battery of evaluation of Amusia. Ann. N.Y. Acad. Sci. 999, 58–75 10.1196/annals.1284.006 14681118

[B46] Phillips-Silver J. Toiviainen P. Gosselin N. Piche O. Nozaradan S. Palmer C. (2011). Born to dance but beat deaf: a new form of congenital amusia. Neuropsychologia 49, 961–969 10.1016/j.neuropsychologia.2011.02.002 21316375

[B47] Repp B. H. (2000). Compensation for subliminal timing perturbations in perceptual-motor synchronization. Psychol. Res. 63, 106–128 10.1007/PL00008170 10946585

[B48] Repp B. H. (2001). Processes underlying adaptation to tempo changes in sensorimotor synchronization. Hum. Mov. Sci. 20, 277–312 10.1016/S0167-9457(01)00049-5 11517673

[B49] Repp B. H. (2005). Sensorimotor synchronization: a review of the tapping literature. Psychon. Bull. Rev. 12, 969–992 10.3758/BF03206433 16615317

[B50] Repp B. H. Keller P. E. (2004). Adaptation to tempo changes in sensorimotor synchronization: effects of intention, attention, and awareness. Q. J. Exp. Psychol. A 57, 499–521 10.1080/02724980343000369 15204138

[B51] Repp B. H. London J. Keller P. E. (2008). Phase correction in sensorimotor synchronization with nonisochronous sequences. Music Percept. 26, 171–175 10.1525/mp.2008.26.2.171

[B52] Schwartze M. Keller P. E. Patel A. D. Kotz S. A. (2011). The impact of basal ganglia lesions on sensorimotor synchronization, spontaneous motor tempo, and the detection of tempo changes. Behav. Brain Res. 216, 685–691 10.1016/j.bbr.2010.09.015 20883725

[B53] Shannon C. E. (1948). A mathematical theory of communication. Bell Syst. Tech. J. 379–423 10.1002/j.1538-7305.1948.tb01338.x

[B54] Shulze H. H. Cordes A. Vorberg D. (2005). Keeping synchrony while tempo changes: accelerando and ritardando. Music Percept. 22, 461–477 10.1525/mp.2005.22.3.461

[B55] Snyder J. Krumhansl C. L. (2001). Tapping to ragtime: cues to pulse finding. Music Percept. 18, 455–489 10.1525/mp.2001.18.4.455

[B56] Sowinski J. Dalla Bella S. (2013). Poor synchronization to the beat may result from deficient auditory-motor mapping. Neuropsychologia 51, 1952–1963 10.1016/j.neuropsychologia.2013.06.027 23838002

[B57] Tass P. Rosenblum M. G. Weule J. Kurths J. Pikovsky P. Volkmann J. (1998). Detection of n:m phase locking from noisy data: application to magnetoencephalography. Phys. Rev. Lett. 81, 3291–3294 10.1103/PhysRevLett.81.3291

[B58] Teki S. Grube M. Griffiths T. D. (2011a). A unified model of time perception accounts for duration-based and beat-based timing mechanisms. Front. Integr. Neurosci 5:90 10.3389/fnint.2011.00090 22319477 PMC3249611

[B59] Teki S. Grube M. Kumar S. Griffiths T. D. (2011b). Distinct neural substrates of duration-based and beat-based auditory timing. J. Neurosci. 31, 3805–3812 10.1523/JNEUROSCI.5561-10.2011 21389235 PMC3074096

[B60] Thompson W. F. (2007). Exploring variants of amusia: tone deafness, rhythm impairment, and intonation insensitivity, in The Inagural International Conference on Music Communication Science. (Sydney).

[B61] Tramo M. J. (2001). Biology and music. Music of the hemispheres. Science 291, 54–56 10.1126/science.10.1126/SCIENCE.1056899 11192009

[B62] Wallentin M. Nielsen H. A. Friis-Olivarius M. Vuust C. Vuust P. (2010). The Musical Ear Test, a new reliable test for measuring musical competence. Learn. Individ. Diff. 20, 188–196 10.1016/j.lindif.2010.02.004

[B63] Wing A. M. Kristofferson A. B. (1973a). Response delays and the timing of discrete motor responses. Percept. Psychophys. 14, 5–12 10.3758/BF0319860711543514

[B64] Wing A. M. Kristofferson A. B. (1973b). The timing of interresponse intervals. Percept. Psychophys. 13, 455–460 10.3758/BF03205802

