# Corrigendum: The Harvard Beat Assessment Test (H-BAT): a battery for assessing beat perception and production and their dissociation

**DOI:** 10.3389/fnhum.2014.00870

**Published:** 2014-11-04

**Authors:** Shinya Fujii, Gottfried Schlaug

**Affiliations:** ^1^Heart and Stroke Foundation Canadian Partnership for Stroke Recovery, Sunnybrook Research InstituteToronto, ON, Canada; ^2^Department of Neurology, Beth Israel Deaconess Medical Center and Harvard Medical SchoolBoston, MA, USA

**Keywords:** rhythm, beat, meter, synchronization, beat-deafness, battery, dissociation

One of the references in this article contained a misspelling of an author name (“Shuit”), which we hereby rectify to “Schuit.”

## Conflict of interest statement

The authors declare that the research was conducted in the absence of any commercial or financial relationships that could be construed as a potential conflict of interest.
